# Before Orchiectomy: Gonadal Function in Testicular Germ Cell Tumors—A Narrative Review

**DOI:** 10.3390/jcm15176857

**Published:** 2026-09-04

**Authors:** Aris Kaltsas, Ilias Giannakodimos, Zisis Kratiras, Nikolaos Sofikitis, Michael Chrisofos

**Affiliations:** 1 Third Department of Urology, Attikon University Hospital, School of Medicine, National and Kapodistrian University of Athens, 12462 Athens, Greece; akaltsas@med.uoa.gr (A.K.); iliasgiannak@med.uoa.gr (I.G.); zkratiras@med.uoa.gr (Z.K.); 2 Department of Urology, Faculty of Medicine, School of Health Sciences, University of Ioannina, 45110 Ioannina, Greece

**Keywords:** testicular germ cell tumors, spermatogenesis, Leydig cells, steroidogenesis, hypogonadism, sperm cryopreservation, fertility preservation, orchiectomy, human chorionic gonadotropin, male infertility

## Abstract

Testicular germ cell tumors (TGCTs) are the most common solid malignancy in young men and are highly curable, making reproductive and endocrine survivorship central concerns. Gonadal dysfunction is often attributed to orchiectomy and gonadotoxic therapy, yet semen and hormonal abnormalities may already be present at diagnosis. This narrative review synthesizes evidence obtained before orchiectomy and, where explicitly identified, broader pretreatment or pre-gonadotoxic evidence. Pre-orchiectomy studies generally report reduced sperm concentration, total sperm count, and progressive motility, together with impaired Sertoli and Leydig cell function. Tumor-derived human chorionic gonadotropin (hCG) can mask reduced Leydig reserve; in hCG-negative men, research-derived testosterone-to-luteinizing hormone and calculated free testosterone-to-luteinizing hormone ratios may aid risk stratification but lack standardized diagnostic cutoffs. Proposed contributors include testicular dysgenesis, contralateral impairment, germ cell neoplasia in situ, local tumor effects, and oxidative or proteomic alterations, although evidential support varies. These findings support fertility counseling at diagnosis, sperm cryopreservation before orchiectomy when feasible without delaying treatment, selected use of onco-microTESE when no usable ejaculate is available, and hCG-aware endocrine follow-up.

## 1. Introduction

Testicular germ cell tumors (TGCTs) are the most common solid malignancy in adolescent and young adult men, with a peak incidence between the ages of 15 and 40 years [1,2]. Although they account for only a small fraction of all male cancers, their incidence has risen steadily across most high-resource populations over recent decades. In the United States, the age-standardized incidence of TGCT increased from 4.71 to 6.22 per 100,000 person-years between 1992 and 2021 [3], and comparable upward trends have been documented across Europe, where approximately one-third of the estimated 74,500 new cases diagnosed worldwide in 2020 occurred [2]. The reasons for this sustained increase remain incompletely understood, but the epidemiological pattern—a rising, predominantly early-onset malignancy of the reproductive years—frames TGCT as a disease whose consequences extend well beyond oncological control.

TGCT is also among the most curable of solid tumors. Advances in platinum-based combination chemotherapy, refined staging, and multidisciplinary management have raised cure rates for advanced disease from roughly 25% in the 1970s to nearly 80%, the highest of any advanced solid tumor, while overall long-term survival now exceeds 95% [1]. As a direct result, the population of long-term survivors continues to grow, and the clinical emphasis has shifted from survival alone toward the quality and durability of that survival. Because most patients are diagnosed during their peak reproductive and hormonally active years and can expect decades of life after treatment, reproductive and endocrine survivorship has become a central, rather than peripheral, concern in the management of this disease [1,2].

Two survivorship domains are particularly consequential: fertility and androgen sufficiency. Impaired spermatogenesis and testosterone deficiency (TD) in men treated for TGCT are conventionally attributed to the definitive interventions themselves—orchiectomy, which removes functional testicular tissue; platinum-based chemotherapy, which is directly gonadotoxic; and radiotherapy, which damages both germinal epithelium and Leydig cells. This treatment-centered model underlies current recommendations for pretreatment fertility counseling and sperm cryopreservation, and it accurately captures an important part of the causal picture [2,4].

That model is, however, incomplete. Clinical and translational evidence indicates that reduced sperm production and subtle Leydig cell insufficiency are often present at diagnosis, before orchiectomy or gonadotoxic therapy. The European Association of Urology accordingly notes that sperm abnormalities and Leydig cell dysfunction are frequently found before orchiectomy and recommends semen preservation, where feasible, ideally before surgery [4]. These observations are consistent with both disease-associated local effects and pre-existing testicular abnormalities, including the testicular dysgenesis syndrome hypothesis, in which impaired spermatogenesis and germ cell malignancy may share a developmental origin [5]. The distinction matters because it advances reproductive and endocrine risk assessment to diagnosis and highlights how tumor-derived human chorionic gonadotropin can obscure reduced Leydig reserve when testosterone is interpreted alone.

This narrative review synthesizes clinical, endocrine, and mechanistic evidence on spermatogenesis and Leydig cell function before orchiectomy and gonadotoxic therapy. It distinguishes strictly pre-orchiectomy evidence from broader pretreatment or pre-gonadotoxic data, examines analytical limitations in baseline endocrine assessment, and considers implications for fertility preservation and survivorship care.

## 2. Materials and Methods

This article was prepared as a narrative review. A structured but non-systematic literature search was conducted in PubMed/MEDLINE and Scopus from database inception to June 2026, supplemented by hand-searching of the reference lists of retrieved articles and of relevant clinical practice guidelines from the European Association of Urology (EAU), the European Society for Medical Oncology–EURACAN (ESMO–EURACAN), the American Society for Reproductive Medicine (ASRM), the American Society of Clinical Oncology (ASCO), and, where relevant, AUA/ASRM male-infertility guidance. Search terms combined Medical Subject Headings and free-text keywords, including “testicular germ cell tumor,” “testicular cancer,” “spermatogenesis,” “semen quality,” “sperm cryopreservation,” “Leydig cell,” “steroidogenesis,” “testosterone,” “luteinizing hormone,” “hypogonadism,” “human chorionic gonadotropin,” “germ cell neoplasia in situ,” “testicular dysgenesis syndrome,” and “fertility preservation,” with emphasis on studies reporting data obtained before orchiectomy or before gonadotoxic therapy where such stratification was available. Evidence was classified as strictly pre-orchiectomy; paired pre- and post-orchiectomy, with both assessments before adjuvant therapy; post-orchiectomy but pre-systemic treatment; or pretreatment or pre-chemotherapy with surgical timing unreported. Accordingly, “pretreatment” and “before chemotherapy” were not treated as synonymous with “before orchiectomy,” and evidence without confirmed preoperative sampling was not used to infer function while the tumor-bearing testis remained in situ.

Eligible sources were restricted to English-language publications and comprised original clinical and translational studies, cohort studies, systematic reviews, meta-analyses, and authoritative guidelines; case reports and small case series were considered only when they addressed otherwise sparsely documented aspects, such as onco-testicular sperm extraction. Titles and abstracts were screened for relevance to pretreatment gonadal function, and full texts were assessed for methodological adequacy and direct pertinence to the scope of the review. Because this work is a narrative synthesis intended to integrate mechanistic and clinical evidence rather than to answer a single quantitative question, formal PRISMA methodology and quantitative data pooling were not applied; studies were selected on the basis of relevance, quality, and representativeness at the authors’ discretion. All quantitative statements were checked against the primary sources. Evidence mapping recorded sampling time, cohort overlap, retrospective design, fertility-preservation referral selection, and assay limitations. All figures were prepared using BioRender AI-assisted functionality and were reviewed and edited by the authors for scientific accuracy.

## 3. Spermatogenesis at Diagnosis: Exocrine Function Before Treatment

Impaired sperm production in men with TGCT is often framed as a consequence of orchiectomy and adjuvant therapy. Yet the exocrine compartment of the testis is frequently compromised at the moment of diagnosis, when semen can be assessed before any intervention has occurred. The underlying studies, their sampling context, and their cohort overlaps are mapped in Table 1.

### 3.1. Semen Parameters in Pretreatment Cohorts

Strictly pre-orchiectomy cohorts consistently show that spermatogenesis is impaired at diagnosis. Petersen et al. documented lower semen quality in men sampled before orchiectomy than in men without testicular cancer [6], and a systematic review of six pre-orchiectomy studies found abnormalities in count, motility, or morphology in every included study [9]. These findings establish that treatment alone cannot explain the baseline deficit, although most cohorts were observational.

The deficit is clinically relevant. In a retrospective cancer sperm-banking cohort of 409 men sampled before cancer therapy, of whom 45% had testicular cancer, sperm density and motility in men with TGCT fell within the intermediate range defined in that study, whereas men with other malignancies were in the fertile range for density; semen quality was statistically lower in men with TGCT than in men with other malignancies, and azoospermic men were not represented [8]. A systematic review identified azoospermia in 57 of 469 men assessed before orchiectomy, while contemporary pre-orchiectomy cohorts report rates of approximately 4.9–11% [10,14,36], compared with about 1% in the general male population [37].

### 3.2. Testicular Germ Cell Tumors Versus Healthy Men and Patients with Other Malignancies

Controlled comparisons suggest that the deficit is not solely a nonspecific cancer effect. In a pre-orchiectomy cohort, total sperm count was lower in men with TGCT than in healthy donors and patients with other malignancies [12]. A separate cancer sperm-banking cohort sampled before cancer therapy likewise found lower density and motility in men with TGCT than in men with other malignancies [8]. Together, these controlled comparisons support a disease-associated deficit already present before surgery.

Clinical modifiers do not fully explain this deficit. In 163 men sampled before orchiectomy, older age, markedly elevated beta-human chorionic gonadotropin, and elevated follicle-stimulating hormone were associated with poorer semen parameters, whereas histology, tumor size, and alpha-fetoprotein were not [13]. Because these factors account for only part of the observed impairment, a broader developmental substrate has been proposed. Testicular dysgenesis syndrome is a fetal-origin hypothesis linking TGCT, impaired spermatogenesis, cryptorchidism, and hypospadias through shared testicular maldevelopment [5]. A separate pre-orchiectomy fertility-preservation cohort also found generally impaired semen parameters without a significant histology association [17].

The effect of clinical stage remains less settled. One pre-orchiectomy cryopreservation cohort found no association between composite stage or histology and semen parameters [10], whereas a subsequent series reported poorer motility and higher rates of asthenozoospermia and teratozoospermia in stage II/III (metastatic) disease [11]. These findings suggest that histology alone is unlikely to explain pretreatment semen impairment, while metastatic burden may contribute in some cohorts.

Interpretation is limited by cohort overlap and referral selection. Several detailed analyses derive from overlapping German cryopreservation databases, and sperm-banking cohorts preferentially include men who accessed or opted for fertility preservation. These datasets should therefore be treated as complementary analyses rather than independent replications. The direction of association is supported across separate cohorts, but population-level prevalence may differ.

### 3.3. Histology of the Tumor-Bearing Testis

Reduced ejaculated sperm output does not imply that the tumor-bearing testis is spermatogenically silent. Histological examination of orchiectomy specimens shows that most cancer-bearing testes retain foci of active spermatogenesis in seminiferous tubules adjacent to and at a distance from the tumor, so the affected gonad often remains a viable source of spermatozoa [27,28]. In a series of 104 tumor-bearing testes, smaller tumor diameter and greater non-cancerous testicular tissue width (NCTW) were positive predictors of preserved spermatogenesis: mature spermatozoa were identified in approximately 93% of specimens with an NCTW of at least 7.5 mm, compared with roughly 41% when the NCTW was narrower, and younger age was likewise favorable [28]. Complementary orchiectomy-specimen studies confirm that a small tumor size favors the presence of complete spermatogenesis, while emphasizing that a larger tumor does not preclude it—active spermatogenesis is present in the majority of cancerous testes regardless of size [27]. These histological observations provide the anatomical rationale for onco-TESE in azoospermic or severely oligozoospermic men. Contralateral impairment is also clinically relevant, reflecting its relationship to GCNIS, testicular dysgenesis, and residual testicular function.

### 3.4. The Impact of Orchiectomy: Pre- Versus Post-Operative Semen Quality

Removal of the tumor-bearing testis can further reduce sperm output. In a two-center retrospective comparison, median total sperm count was 56.9 × 10^6^ before versus 13 × 10^6^ shortly after orchiectomy, and azoospermia was present in 4.9% versus 14.9% [14]. Because the preoperative and postoperative groups comprised different men, this study supports a timing association rather than an individual causal change.

Paired studies provide stronger within-person evidence. In an older cohort, sperm concentration declined in 30 of 35 men, and azoospermia developed in 3, accompanied by higher follicle-stimulating hormone and lower inhibin B [19]. Recent prospective paired cohorts similarly reported postoperative reductions in concentration, total count, and/or progressive motility, although not every parameter declined significantly in every study, and sperm DNA fragmentation sometimes improved [15,16]. These findings favor pre-orchiectomy banking when feasible while allowing additional post-orchiectomy banking in selected men before gonadotoxic therapy.

## 4. Leydig Cell Steroidogenesis and Endocrine Phenotypes at Diagnosis

The endocrine compartment of the testis is affected at diagnosis alongside the exocrine one, but its impairment is more easily overlooked. Leydig cell dysfunction in TGCT is typically compensated rather than overt: luteinizing hormone (LH) rises to maintain testosterone output, so total testosterone may remain within the normal range while the underlying steroidogenic reserve is already reduced. Detecting this state requires looking beyond a single testosterone value—and is further complicated by tumor-derived human chorionic gonadotropin, which can stimulate the Leydig cells and mask the deficit.

### 4.1. Prevalence of Pre-Orchiectomy Leydig Cell Dysfunction

Compensated Leydig cell dysfunction may be missed when testosterone is interpreted alone. In a case–control study of 561 patients and 561 controls, Bandak et al. calculated total testosterone-to-LH and calculated free testosterone-to-LH ratios and defined abnormal values using bivariate charts derived from the controls [7]. Among 374 hCG-negative patients, 25% and 24%, respectively, fell outside those control-derived distributions. These estimates identify a research-defined high-risk phenotype; the ratios lack harmonized, assay-specific cutoffs and are not validated diagnostic tests for routine care.

Contralateral germ cell neoplasia in situ, increasing age, and larger tumor size were associated with an abnormal calculated free testosterone-to-LH ratio [7]. These associations are consistent with field-wide developmental impairment and local parenchymal effects but do not establish causality. A separate cohort in which higher pre-orchiectomy LH and follicle-stimulating hormone accompanied larger tumors provides supportive, not confirmatory, evidence [21].

Exclusion of hCG-positive patients was essential because tumor-derived hCG can raise testosterone and suppress LH, thereby inflating either ratio. Bandak et al. hypothesized, but did not demonstrate longitudinally, that pre-existing dysfunction may predict post-treatment testosterone deficiency [7].

### 4.2. The Masking Effect of Human Chorionic Gonadotropin

Human chorionic gonadotropin shares the LH receptor and acts as a potent functional analog of LH on the Leydig cell. In germ cell tumors that secrete hCG—a substantial subset, particularly non-seminomas and any tumor with a choriocarcinomatous component—this tumor-derived stimulation drives Leydig cell production of testosterone and, through aromatization, estradiol, while the resulting negative feedback suppresses pituitary secretion of LH and FSH. In a study of men with disseminated testicular cancer, higher serum β-hCG was accordingly associated with higher testosterone, estradiol, and prolactin and with lower LH and FSH, alongside poorer semen quality [20]. The clinically important consequence is that a man with genuinely reduced Leydig cell reserve may present at diagnosis with a normal—or even elevated—serum testosterone and a suppressed LH, giving a spurious impression of intact endocrine function.

The orchiectomy transition provides direct evidence of this artifact: removal of an hCG-secreting tumor is followed by lower testosterone and estradiol and higher gonadotropins as the stimulated state resolves [19]. Accordingly, hCG-positive and hCG-negative groups should be analyzed separately [6]. Total testosterone-to-LH and calculated free testosterone-to-LH ratios have been used for research-based risk stratification only in hCG-negative men; hCG simultaneously raises testosterone and suppresses LH, making either ratio uninterpretable when hCG is elevated [7].

At diagnosis, normal or high testosterone does not exclude reduced Leydig reserve when hCG is elevated. If a complete endocrine panel cannot be obtained before urgent orchiectomy, cancer treatment should not be delayed; available preoperative results should be recorded, and morning testosterone, SHBG, LH, and FSH may be completed or repeated after surgery and clinical recovery, preferably before subsequent gonadotoxic therapy when feasible [7,19]. When preoperative hCG is elevated, LH-dependent indices should be deferred until hCG is no longer elevated; neither reassessment nor hCG clearance should delay indicated systemic therapy. No guideline establishes a fixed postoperative reassessment interval. In hCG-negative men, the ratios may add context to risk stratification but should not be used alone to diagnose hypogonadism because standardized cutoffs are unavailable.

### 4.3. Preoperative Hormonal Subsets

The recognition that endocrine and exocrine abnormalities coexist at diagnosis is long-standing. In an early series that profiled both compartments before orchiectomy, men with testicular tumors were shown to have concurrent disturbances of reproductive hormones and semen quality, establishing that the endocrine derangement is a feature of the disease at presentation rather than solely a consequence of its treatment [18]. What that early work could not fully capture—because it predated large multicenter datasets and modern clustering methods—was the heterogeneity of the endocrine phenotype across patients.

A contemporary multicenter study measured preoperative LH, FSH, testosterone, estradiol, and prolactin in 518 men with testicular germ cell cancer; the complete-case latent-class analysis included 422 and identified three hormonal profiles [22]. Estradiol was above the reference range in approximately 16% and below it in 32%, and levels were higher in non-seminoma than seminoma. These patterns may reflect hCG-driven aromatization or, in some cases, possible tumor steroid production, but the cross-sectional data do not establish a mechanism [22].

Because endocrine phenotypes vary across patients, a single testosterone measurement cannot represent the state at diagnosis; a multi-analyte baseline including LH, FSH, testosterone, and hCG is more informative, with estradiol and prolactin added when clinically indicated [22]. Estradiol excess may manifest as gynecomastia but is not a standard surveillance marker. Conversely, isolated low-level hCG with low testosterone and elevated or non-suppressed LH may reflect immunoassay cross-reactivity rather than biologically active tumor-derived hCG [22].

### 4.4. Disruption of the Hypothalamic–Pituitary–Gonadal Axis

These endocrine abnormalities are best understood not in isolation but as perturbations of a single feedback system. In the intact axis, hypothalamic gonadotropin-releasing hormone drives pituitary secretion of LH and FSH; LH stimulates Leydig cell testosterone production, while FSH, together with high intratesticular testosterone, supports Sertoli cell and germinal function; testosterone and inhibin B in turn provide negative feedback on LH and FSH, respectively. In men with TGCT, this loop is disturbed at several nodes simultaneously and already before treatment, producing the composite signature summarized in Table 2.

Table 2 shows that apparently discordant patterns are largely explained by germinal or Sertoli-cell impairment, compensated Leydig function, and hCG-mediated feedback. Baseline assessment is therefore multi-analyte and hCG-aware; ratio-based interpretations remain nonstandardized.

## 5. Mechanistic Insights

**Figure 1:**
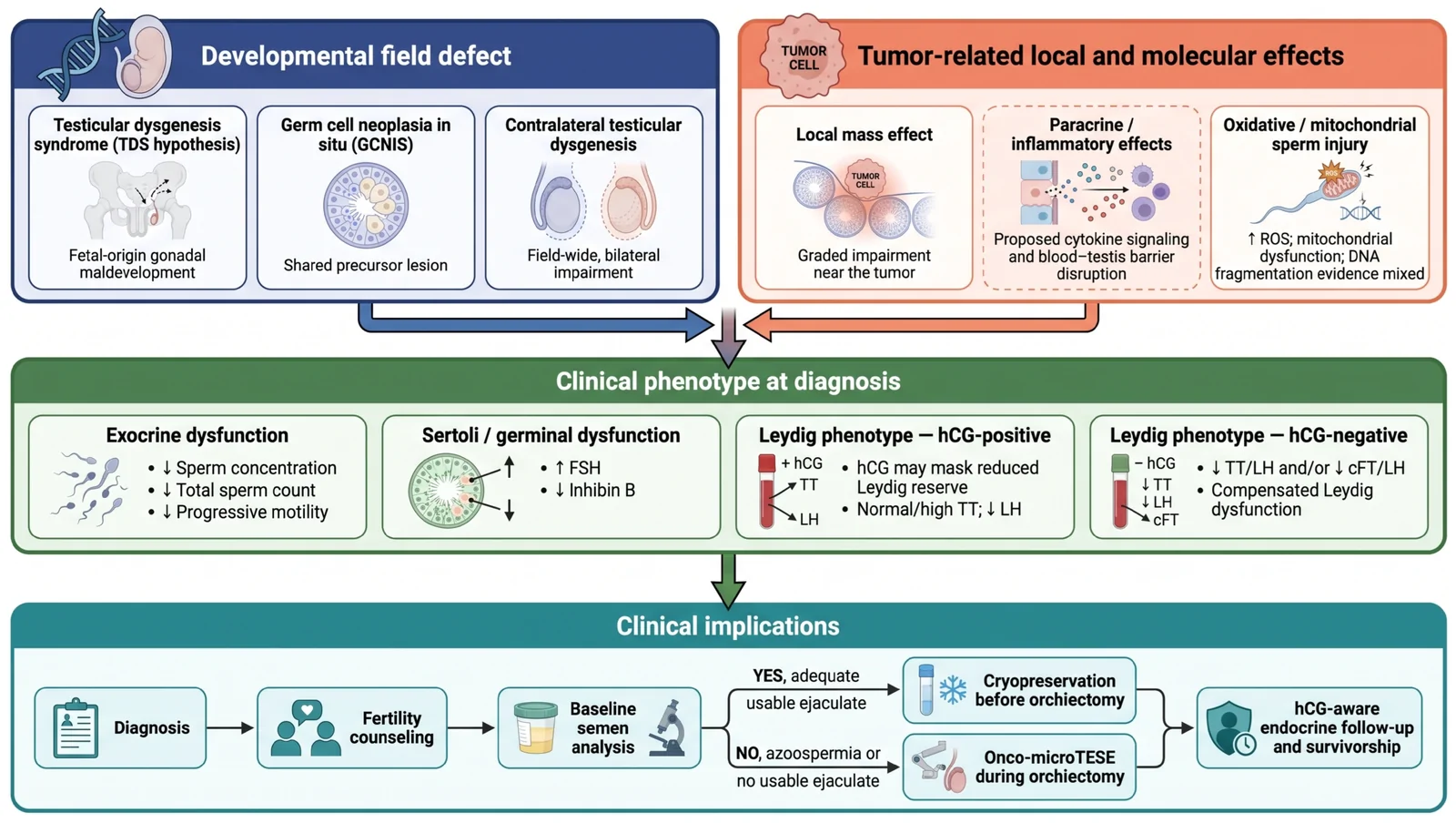
Integrative model of pretreatment gonadal dysfunction in TGCT. Developmental field defects and tumor-related local or molecular effects may converge on exocrine, Sertoli or germinal, and Leydig-cell phenotypes at diagnosis. Evidence is comparatively stronger for developmental and GCNIS associations and for hCG-mediated masking of Leydig function; local mass effects have spatial histological support, whereas paracrine, inflammatory, and autoimmune mechanisms and candidate molecular markers remain proposed or exploratory, as indicated by the dashed outline. Contralateral and field-wide involvement applies to a subset of patients and does not imply universal bilateral disease. In hCG-negative men, reduced Leydig reserve is typically compensated: total and calculated free testosterone fall while LH rises, lowering the TT/LH and cFT/LH ratios; in hCG-positive men, tumor-derived hCG raises testosterone and suppresses LH, so both ratios are uninterpretable. cFT, calculated free testosterone; DNA, deoxyribonucleic acid; FSH, follicle-stimulating hormone; GCNIS, germ cell neoplasia in situ; hCG, human chorionic gonadotropin; LH, luteinizing hormone; microTESE, microdissection testicular sperm extraction; ROS, reactive oxygen species; TDS, testicular dysgenesis syndrome; TT, total testosterone. Within boxes, ↑ and ↓ denote an increase or a decrease in the parameter indicated. Created in BioRender. Kaltsas, A. (2026) https://BioRender.com/st3ie7j. BioRender AI-assisted functionality was used during figure preparation; the scientific content was reviewed and approved by the authors.

Several mechanisms may contribute to gonadal dysfunction at TGCT diagnosis, but their evidential support is unequal. Developmental abnormalities, germ cell neoplasia in situ, local effects on adjacent parenchyma, and oxidative or proteomic changes may overlap rather than represent a single causal pathway. These proposed relationships are summarized in Figure 1.

### 5.1. Testicular Dysgenesis Syndrome and a Common Fetal Origin

The testicular dysgenesis syndrome hypothesis proposes that poor semen quality, testicular germ cell cancer, cryptorchidism, and hypospadias may arise as different manifestations of disturbed fetal testis development under interacting genetic and environmental influences [5]. It therefore offers a developmental framework for the coexistence of impaired spermatogenesis and TGCT, supported by broader epidemiological and biological synthesis [38].

Cryptorchidism and subfertility are established TGCT risk factors, and histological abnormalities in men with TGCT are consistent with a dysgenetic substrate [5,38]. However, these associations do not prove that TDS accounts for the semen and endocrine abnormalities of every patient.

The strength of this evidence should not be overstated. TDS is a unifying hypothesis with partial explanatory scope; it does not imply that every affected man develops all four component disorders, and many cases of isolated impaired spermatogenesis have heterogeneous or postnatal causes [39]. In men with TGCT, TDS therefore offers one plausible framework for the coexistence of impaired spermatogenesis and Leydig function, but it does not establish that either abnormality has a single developmental cause.

### 5.2. Germ Cell Neoplasia In Situ and Contralateral Impairment

A related histological finding is germ cell neoplasia in situ (GCNIS), the precursor of nearly all postpubertal-type TGCTs other than spermatocytic tumors and prepubertal-type germ cell tumors [40]. GCNIS is associated with a dysgenetic tubular environment and impaired spermatogenesis, although it should not be assumed to explain all functional abnormalities at diagnosis.

Dysgenetic abnormalities may also involve the contralateral testis. In a biopsy series of 218 men with testicular germ cell cancer, at least one dysgenetic histological feature was identified in 25.2% [26]. This finding supports a field-defect hypothesis in a subset of patients but does not imply universal bilateral abnormality. Together with the recognized risk of contralateral GCNIS, it cautions against assuming that the remaining testis is functionally intact in every patient [2,4,26].

The relevance of contralateral pathology extends to the endocrine compartment. Contralateral germ cell neoplasia in situ was independently associated with pre-orchiectomy Leydig cell dysfunction [7], and an older post-orchiectomy, pre-gonadotoxic cohort found Leydig dysfunction in 11 of 24 men with contralateral carcinoma in situ versus 2 of 30 without it, together with lower sperm concentration [25]. These findings link GCNIS, impaired spermatogenesis, contralateral involvement, and endocrine impairment in a subset of patients, but they cannot explain all baseline gonadal dysfunction.

### 5.3. Local Tumor, Paracrine, and Inflammatory Effects

Distinct from the developmental mechanisms above, the established tumor exerts local effects on the surrounding testis. The clearest evidence is histological and spatial. In a review of orchiectomy specimens, impairment of ipsilateral spermatogenesis was most marked in the seminiferous tubules immediately adjacent to the tumor and lessened with increasing distance from it, indicating a graded local effect superimposed on any diffuse impairment [23]. This graded pattern is consistent with the histological predictors noted earlier and supports a local mass/paracrine effect superimposed on diffuse dysgenetic impairment. Notably, a comparable zone of impaired spermatogenesis adjacent to the tumor is seen with non-germ-cell testicular tumors as well [24], which argues that at least part of this effect is a nonspecific consequence of an expanding intratesticular mass rather than a process unique to germ cell neoplasia.

The mediators of this local effect are less firmly established and are best regarded as proposed rather than proven contributors in the pretreatment setting. Plausible mechanisms include physical compression and pressure atrophy of adjacent tubules, disruption of the local microvasculature, and paracrine effects of factors and cytokines secreted within the tumor microenvironment [9]. A related consideration is immunological: the testis is an immune-privileged organ whose privilege depends on the blood–testis barrier formed by adjacent Sertoli cells, and disruption of this barrier by an invasive or expanding tumor could expose sequestered germ-cell antigens, provoking a local inflammatory or autoimmune response—including anti-sperm antibodies—that further impairs spermatogenesis, while inflammatory cytokines may additionally suppress Leydig cell steroidogenesis. These immunological and paracrine mechanisms are biologically coherent and consistent with general testicular pathophysiology, but direct evidence specifically in men with untreated TGCT is limited, and they should be interpreted as hypotheses that plausibly complement, rather than replace, the better-documented mass effect [9].

Developmental and local explanations are complementary hypotheses operating at different scales. TDS and GCNIS may be associated with field-wide or bilateral abnormalities in a subset of patients, whereas spatial gradients in orchiectomy specimens support a more proximate ipsilateral tumor effect [5,23,26]. Pre-existing or contralateral vulnerability could therefore coexist with local disruption around the tumor, but the available cross-sectional histology cannot establish temporal sequence or reversibility. The frequent preservation of spermatogenesis in tissue distant from the tumor provides an anatomical rationale for onco-testicular sperm extraction [27,28].

### 5.4. Oxidative Stress, Sperm DNA Fragmentation, Proteomic and Mitochondrial Alterations

Molecular abnormalities have been detected in sperm obtained before gonadotoxic therapy. One small proteomic study reported lower NDUFS1 and higher CD63 expression in both normozoospermic and asthenozoospermic TGCT samples [30]. A second 15-patient non-seminoma cohort replicated lower NDUFS1 and also reported lower UQCRC2 and ATP1A4 [31]. The partial replication supports mitochondrial involvement, but both studies were small and provide no standardized clinical cutoffs or reproductive-outcome validation.

Oxidative and DNA-integrity data are heterogeneous. Flow-cytometric analysis found higher viable-sperm oxidative stress and DNA fragmentation in mixed cancer cohorts that included TGCT [32], whereas a review of 11 sperm chromatin structure assay studies found increased pretreatment fragmentation in 7 studies but not in 4 [29]. A paired study also reported lower sperm DNA fragmentation after orchiectomy despite lower conventional semen parameters [16]. These discordant results, together with assay and cohort differences, show that semen parameters and DNA integrity are related but non-interchangeable endpoints.

These molecular findings remain exploratory. NDUFS1 and CD63 expression and quantitative sperm oxidative stress lack standardized thresholds, independent clinical validation, and evidence of incremental value for reproductive counseling. They should not be integrated into routine counseling until prospectively validated against clinically meaningful reproductive outcomes [30,31,32].

## 6. Clinical Implications

**Figure 2:**
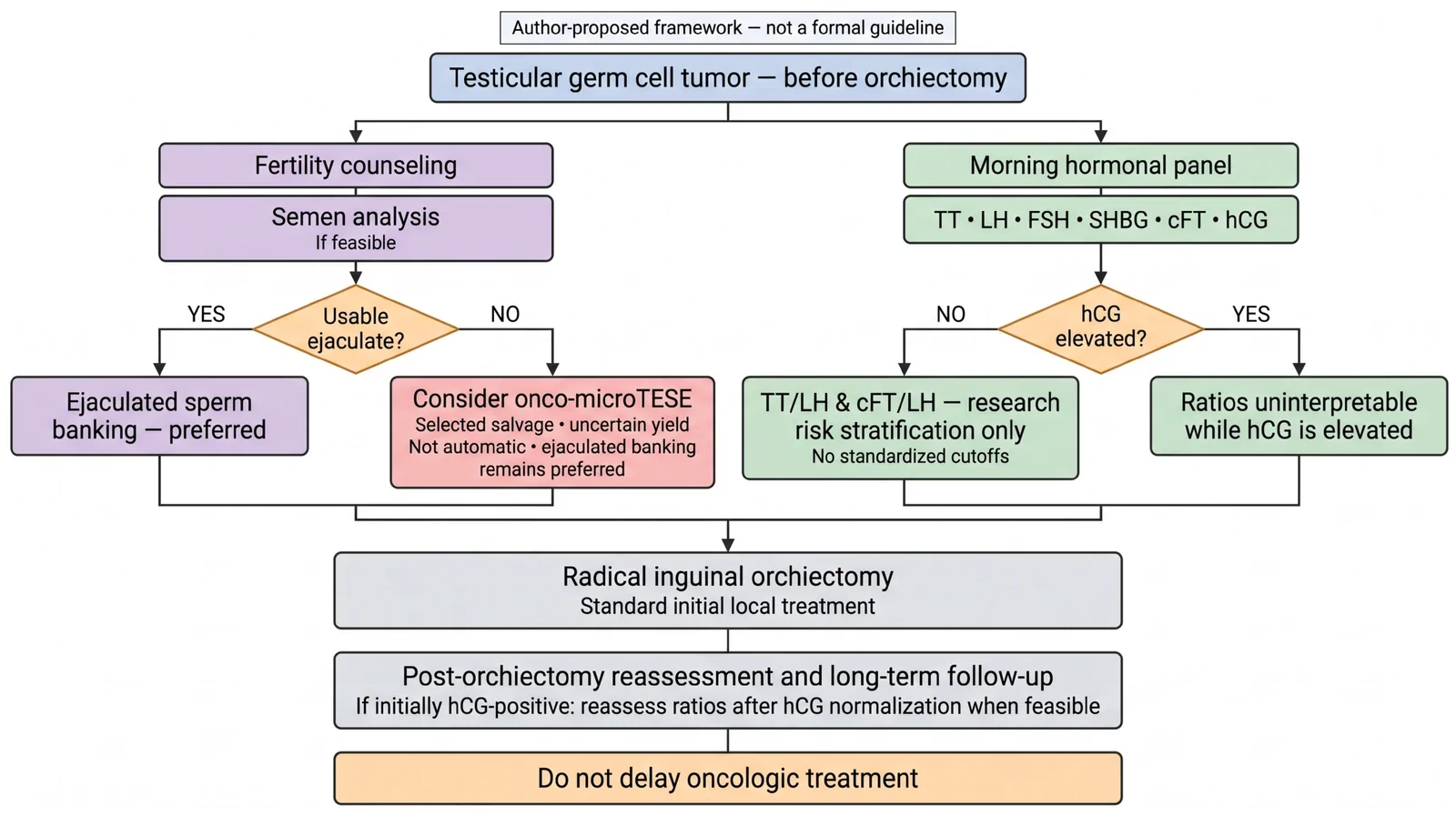
Author-proposed clinical framework for pretreatment reproductive and endocrine assessment in men with testicular germ cell tumors; this narrative synthesis is not a formal guideline algorithm. Fertility counseling with semen analysis and a baseline morning hormonal panel proceed in parallel when feasible, without delaying oncologic treatment. Ejaculated cryopreservation is preferred before orchiectomy; one to three samples may be collected according to urgency and semen quality. Onco-microTESE is a selected salvage strategy for azoospermia, severe oligoasthenoteratozoospermia, or no usable ejaculate; it is not automatic and does not replace ejaculated banking. In hCG-negative men, TT/LH and cFT/LH may support research-based risk stratification but lack standardized diagnostic cutoffs; while hCG is elevated, both ratios are uninterpretable, and reassessment after hCG normalization is preferred when feasible. Inhibin B, estradiol, or prolactin may be added to the baseline panel when clinically indicated. cFT, calculated free testosterone; FSH, follicle-stimulating hormone; hCG, human chorionic gonadotropin; LH, luteinizing hormone; microTESE, microdissection testicular sperm extraction; SHBG, sex hormone-binding globulin; TT, total testosterone. Created in BioRender. Kaltsas, A. (2026) https://BioRender.com/yssxvtu. BioRender AI-assisted functionality was used during figure preparation; the scientific content was reviewed and approved by the authors.

The evidence has three practical implications: fertility counseling and the timing of sperm banking, selected surgical sperm retrieval when no usable ejaculate is available, and hCG-aware endocrine assessment integrated into survivorship care. Figure 2 presents an author-proposed clinical framework derived from this narrative synthesis; it is not a formal guideline algorithm.

### 6.1. Fertility Counseling and Sperm Banking Before Orchiectomy

Fertility counseling should occur at diagnosis. Current guidance supports discussing reproductive risk with all postpubertal patients and offering sperm cryopreservation as the established first-line option [4,41,42,43]. The recommendation is particularly important for a solitary testis, bilateral tumors, or an abnormal contralateral testis, but the opportunity should not be restricted to these higher-risk groups.

When oncologically feasible, ejaculated sperm should be banked before orchiectomy because semen quality is often better before than shortly after surgery [4,14,15,16,41]. The American Society of Clinical Oncology likewise recommends offering sperm cryopreservation before cancer-directed treatment and considering testicular sperm extraction when a semen sample cannot be provided [43]. One to three samples may be collected according to urgency, feasibility, and semen quality; definitive treatment must not be inappropriately delayed.

Implementation remains a major gap. In a US claims cohort of 5907 patients aged 16–49 years who underwent orchiectomy, documented fertility-preservation counseling and sperm cryopreservation within 180 days of diagnosis occurred in 9.53% and 8.43%, respectively; pre-orchiectomy rates were 4.18% and 4.84% [44]. Perceived urgency, referral access, cost, and incomplete documentation may all contribute. Rapid referral pathways, same-day or expedited banking, patient navigation, transparent cost counseling, and standardized documentation are practical responses, although their implementation effectiveness requires prospective evaluation.

### 6.2. Onco-TESE and Onco-MicroTESE for Azoospermic Patients

Onco-microTESE is a selected perioperative salvage strategy for men with azoospermia, severe oligoasthenoteratozoospermia, or no usable ejaculated sperm after banking has been attempted when feasible. It is most often performed during radical inguinal orchiectomy by ex vivo microdissection of non-tumorous tissue from the affected testis, with contralateral rescue considered only when appropriate [34]. It is not a replacement for ejaculated cryopreservation.

Retrieval rates vary with selection and technique. Sperm were retrieved in 3 of 9 azoospermic men in one series and in about one quarter of 38 azoospermic men in another [34,35]. No baseline variable reliably predicts success, and intracytoplasmic sperm injection is required when sperm are recovered. Counseling should therefore present uncertain yield without allowing the procedure to compromise oncologic timing.

Delivery requires coordination among urology, andrology, and embryology teams. Ejaculated banking should be attempted first when possible; consent should address uncertain yield, the need for intracytoplasmic sperm injection, and the circumstances in which contralateral rescue might be considered. The procedure should be performed at orchiectomy in experienced centers and must not delay definitive cancer treatment [33,34,35].

### 6.3. Baseline Endocrine Assessment and Survivorship

When feasible, baseline endocrine assessment should include morning total testosterone, LH, FSH, SHBG for calculated free testosterone, and hCG, with inhibin B, estradiol, or prolactin added when clinically indicated. Testosterone should be interpreted with LH and hCG. In hCG-negative men, TT/LH or cFT/LH ratios may support risk stratification, but they are not standardized diagnostic tests [7]. If surgery is urgent, the panel can be completed or repeated postoperatively as described above without delaying orchiectomy.

Survivorship data support continued endocrine monitoring. The odds of testosterone deficiency are higher after standard chemotherapy and rise further with more intensive treatment [45], while longitudinal cohorts show declining testosterone and rising gonadotropins with age [46,47]. Hypogonadism may compound the metabolic and cardiovascular morbidity observed in testicular cancer survivors, particularly after platinum-based chemotherapy [48,49,50].

Long-term care should monitor morning testosterone together with symptoms and address metabolic and cardiovascular risk. Testosterone replacement should be considered only for symptomatic, biochemically confirmed hypogonadism in accordance with established guidance. Exogenous testosterone should be avoided in men with current or future fertility goals because it suppresses gonadotropins and spermatogenesis [51].

## 7. Future Directions

Future studies should prioritize prospective, paired within-patient sampling before orchiectomy, after orchiectomy but before gonadotoxic therapy, and during long-term follow-up. This design would reduce the selection and between-group biases of retrospective sperm-banking cohorts and better separate disease-associated abnormalities from acute surgical and later treatment effects [14,15,16].

Endocrine and molecular markers also require validation. Assay-specific reference distributions and external validation are needed before TT/LH or cFT/LH can move beyond research-based risk stratification [7]. Candidate sperm proteins such as NDUFS1 and CD63 require standardized methods, clinical cutoffs, and prospective association with paternity or live-birth outcomes before they can inform counseling [30,31].

Implementation research should test rapid referral networks, patient navigation, cost-support pathways, and standardized documentation against measurable improvements in pre-orchiectomy counseling and cryopreservation, particularly outside academic centers and in underserved groups [44].

Cross-study comparability remains limited by changing World Health Organization lower fifth-percentile semen reference distributions across manual editions [52,53], differences in hormone-assay platform and calibration [54], control-derived ratio thresholds, conflicting sperm DNA fragmentation results, hCG-related endocrine confounding, cohort overlap, and reliance on semen or hormonal surrogates rather than reproductive outcomes. These limitations require cautious interpretation of prevalence estimates and mechanistic claims.

## 8. Conclusions

Evidence from pre-orchiectomy cohorts indicates that impaired semen quality and compensated Leydig cell dysfunction are common at diagnosis, although much of the literature is retrospective and selectively sampled. The findings are consistent with overlapping developmental, contralateral, local tumor, and molecular contributors; they do not establish a single causal gonadal pathology. Clinically, fertility counseling and ejaculated sperm cryopreservation should be offered before orchiectomy when feasible and without delaying cancer treatment, while onco-microTESE is a selected salvage option when no usable ejaculate is available. Baseline endocrine evaluation should be hCG-aware. Research-derived TT/LH and cFT/LH ratios may support risk stratification in hCG-negative men but are not standardized diagnostic tests. Reproductive and endocrine follow-up should begin at diagnosis and continue through survivorship.

## Figures and Tables

**Table 1:** Evidence map of studies informing pretreatment spermatogenic and Leydig-cell dysfunction in men with testicular germ cell tumors.

Study (Year) [Ref]	Design; *n*	Timing	Key Findings	Caveats/Cohort Overlap
**A. Pretreatment semen quality and orchiectomy timing**				
Petersen 1999 [6]	Pre-orchiectomy cohort; 83 enrolled (semen *n* = 63; hormones *n* = 71)	Pre-orchiectomy	Reduced semen quality before any definitive treatment	hCG-positive analyzed separately; older assays; possible overlap with later Copenhagen data [7].
Williams 2009 [8]	Retrospective sperm-banking cohort; 409 men (45% testicular cancer)	Before cancer therapy; pre-orchiectomy status not specified	TGCT density and motility in the intermediate range; other malignancies in the fertile range for density	Sperm-banking selection; mixed malignancies; azoospermic men not represented. Independent US cohort.
Djaladat 2014 [9]	Systematic review; 6 studies, 135 pre-orchiectomy patients (701 records screened)	Pre-orchiectomy (secondary synthesis)	Every included study: abnormal count, motility, or morphology before orchiectomy	Secondary synthesis—not an independent cohort.
Badia 2023 [10]	Pre-orchiectomy cryopreservation cohort; 38	Pre-orchiectomy	No association of clinical stage or histology with semen parameters	Small; underpowered for subgroups. Independent US; contrasts Mendes on stage.
Mendes 2024 [11]	Pre-orchiectomy cryopreservation cohort; 64 (33 seminoma/31 non-seminoma)	Pre-orchiectomy	Stage II/III: poorer motility, more astheno-/teratozoospermia; histology no effect	Retrospective; modest *n*. Independent Portuguese; contrasts Badia on stage.
Ruf 2025 [12]	Pre-orchiectomy with donor and other-malignancy controls; 664 (163 TGCT/289 donors/212 other)	Pre-orchiectomy	Median total sperm count lower in TGCT than donors and other malignancies (disease-specific)	Shares the 163-patient Hamburg pre-orchiectomy cohort with [13] and the preoperative arm of [14].
Dieckmann 2025 [13]	Retrospective clinical-factor analysis; 163 TGCT	Pre-orchiectomy	Older age → lower volume/motility; greatly ↑ β-hCG and ↑ FSH → lower total sperm count	Same 163 Hamburg men as [12,14], different analysis.
Dieckmann 2025 [14]	Two-center timing comparison; 163 preoperative vs. 242 postoperative	Pre- vs. post-orchiectomy (between-group comparison)	Preoperative semen superior; azoospermia rose 4.9% → 14.9% after orchiectomy	Different patients pre/post. Pre-op arm = same 163 Hamburg cohort as [12,13].
Tang 2026 [15]	Prospective paired before/after; 25 TGCT	Paired pre-/post-orchiectomy	↓ Concentration and progressive motility after orchiectomy; total count not significantly ↓	Small; addresses the paired-design gap of [14]. Independent prospective cohort.
Buonacquisto 2026 [16]	Prospective paired before/after incl. SDF; 176 (SDF subset 48)	Paired pre-/post-orchiectomy	↓ Total count and progressive motility after orchiectomy; SDF decreased after orchiectomy	SDF is a surrogate endpoint. Independent Italian (Rome) cohort.
Cariati 2026 [17]	Retrospective fertility-preservation cohort; 278 analyzed (abstract reports 284) + 51 controls	Strictly pre-orchiectomy and pre-gonadotoxic	Parameters predominantly below WHO percentiles; no histology–semen association	Referral selection; analytic *n* = 278 despite abstract *n* = 284. Independent Italian cohort.
**B. Endocrine and Leydig-cell evidence at diagnosis**				
Carroll 1987 [18]	Early endocrine + exocrine profiling; 15	Pre-orchiectomy	Concurrent semen and reproductive-hormone abnormalities before treatment	Old assays; pre-hCG-aware era. Independent historical cohort.
Petersen 1999 [19]	Paired pre/post endocrine + semen; 48 (semen in 35)	Paired pre-/post-orchiectomy	Concentration ↓ in 30/35; new azoospermia 3; ↑ FSH, ↓ inhibin B; T/E2 fell after hCG-source removal	Small; older assays; 48-patient follow-up subset of [6], not an independent cohort.
de Bruin 2009 [20]	Semen + hormones in disseminated disease; 107 stored/62 analyzable	Pre-chemotherapy; orchiectomy timing not reported	↑ β-hCG → ↑ T, E2, prolactin and ↓ LH/FSH with poorer semen (masking)	Metastatic-only; hCG confounds Leydig estimate. Independent Dutch—hCG-masking evidence.
Bandak 2017 [7]	Case–control, TT/LH and cFT/LH ratios; 561 + 561 (374 hCG-negative analyzed)	Pre-orchiectomy	Research-defined abnormal ratios in ~24–25%; associated with contralateral GCNIS, age, and tumor size	Control-derived, nonharmonized ratios; hCG-positive patients not interpretable; cohort overlap uncertain.
Pineault 2021 [21]	Retrospective hormone–pathology association; 52	Pre-orchiectomy	Higher pre-orchiectomy LH and FSH accompanied larger germ-cell tumors	No paired semen; no validated cutoffs. Independent US; supportive.
Törzsök 2023 [22]	Multicenter pre-surgery hormones; 518 total (latent-class complete-case *n* = 422)	Pre-orchiectomy	Three hormonal subsets; E2 abnormal in ~half, higher in NSGCT; supports multi-analyte panel	Cluster analysis needs external validation. Independent multicenter cohort; complements [7].
**C. Histological, contralateral, local and molecular mechanisms**				
Ho 1992 [23]	Ipsilateral spermatogenesis in orchiectomy specimens; 28	Post-orchiectomy specimen	Impairment greatest adjacent to tumor, lessening with distance (local effect)	Surrogate; cannot fully separate developmental vs. local. Independent.
Ho 1994 [24]	Comparator: non-germ-cell tumors; 20 malignant and 15 benign	Post-orchiectomy specimen	Similar peritumoral impairment with non-GCT indicates that the local effect is not GCT-specific	Not TGCT; comparator only—not an independent baseline cohort.
Petersen 1999 [25]	Cross-sectional by contralateral CIS/GCNIS; 54 (24 vs. 30)	Post-orchiectomy, pretreatment	Contralateral CIS/GCNIS → Leydig dysfunction 11/24 vs. 2/30 and lower sperm concentration	Small; CIS terminology predates GCNIS; possible Copenhagen cohort overlap.
Hoei-Hansen 2003 [26]	Contralateral-biopsy histology; 218	At orchiectomy (contralateral biopsy)	At least one dysgenetic feature identified in 25.2% of contralateral biopsies	Histological surrogate; field-defect evidence applies to a subset.
Choy 2013 [27]	Orchiectomy-specimen histology; 83 (77 cancerous; 41 seminoma/36 non-seminoma)	Post-orchiectomy specimen	Active spermatogenesis in most cancerous testes → anatomic rationale for onco-TESE	Histological surrogate; no ejaculate/ART outcomes. Independent US cohort.
Suzuki 2015 [28]	Orchiectomy-specimen histology; 104 tumor-bearing testes	Post-orchiectomy specimen	Spermatozoa in ~93% when NCTW ≥ 7.5 mm versus ~41% when narrower; smaller tumor favorable	Surrogate. Independent Japanese cohort; supports ex vivo onco-TESE.
Paoli 2018 [29]	Narrative review of sperm DNA damage; 11 SCSA studies	Mixed	7/11 studies ↑ pretreatment SDF, 4 did not (heterogeneous)	Secondary synthesis—not an independent cohort.
Panner Selvam 2019 [30]	Sperm proteomics; 31 TGCT (20 normo/11 astheno) + 9 controls	Pre-gonadotoxic	↓ NDUFS1 and ↑ CD63 even in normozoospermic TGCT sperm	Discovery proteomics; not clinically validated. Continuity with [31].
Dias 2020 [31]	Proteomic validation, non-seminoma; 15 + 15 controls	Pre-gonadotoxic	Replicated ↓ NDUFS1; also ↓ UQCRC2 and ↓ ATP1A4 (mitochondrial dysfunction)	Small; no clinical thresholds. Methodologic continuity with [30].
Calamai 2023 [32]	Flow-cytometry oxidative stress/SDF; 85–96 cancer (mixed testicular and hematological)	Pre-gonadotoxic; mixed pre-/post-orchiectomy	Viable-sperm oxidative stress several-fold higher; ↑ SDF; OS tended higher pre- than post-orchiectomy	Mixed-cancer; TGCT subgroup to verify. Independent translational cohort.
**D. Surgical sperm retrieval and salvage fertility preservation**				
Ogouma 2022 [33]	Systematic review of TESE in malignancy; 34 articles (15 onco-TESE)	Pre/post-treatment	TESE feasible in malignant disease, including onco-TESE at orchiectomy	Heterogeneous; secondary synthesis—not independent baseline evidence.
Cirigliano 2023 [34]	Single-center onco-microTESE and literature; 9 azoospermic/severe	At orchiectomy (selected salvage)	Sperm retrieved in 3/9; ICSI pregnancies reported	Very small, unpredictable; needs microsurgery/embryology. Independent Italian cohort.
Flores 2025 [35]	Retrospective onco-TESE at radical orchiectomy; 38 azoospermic	At orchiectomy (selected salvage)	Retrieval successful in ~25%; no baseline variable reliably predictive	Selected azoospermic men. Independent US surgical cohort.

Studies are grouped by evidence type; reviews and mechanistic/comparator studies are not independent clinical cohorts. Overlapping German (Hamburg) cohorts are made explicit. ART, assisted reproductive technology; cFT, calculated free testosterone; CIS, carcinoma in situ; DNA, deoxyribonucleic acid; E2, estradiol; FSH, follicle-stimulating hormone; GCNIS, germ cell neoplasia in situ; hCG, human chorionic gonadotropin; ICSI, intracytoplasmic sperm injection; LH, luteinizing hormone; microTESE, microdissection testicular sperm extraction; NCTW, non-cancerous testicular tissue width; NSGCT, non-seminomatous germ cell tumor; OS, oxidative stress; SCSA, sperm chromatin structure assay; SDF, sperm DNA fragmentation; T, testosterone; TESE, testicular sperm extraction; TGCT, testicular germ cell tumor; TT, total testosterone; WHO, World Health Organization. Symbols: ↑ and ↓ denote a reported increase or decrease in the parameter indicated; → denotes a reported association or the inference drawn from it, not a demonstrated causal effect, and, where two values are given, a change from the first value to the second.

**Table 2:** Characteristic hypothalamic–pituitary–gonadal findings at diagnosis in men with testicular germ cell tumors, before orchiectomy or gonadotoxic therapy.

Marker	Typical Change at Diagnosis	Principal Driver	Interpretive Caveat/Evidence
Testosterone (TT)	Normal or low-normal; occasionally spuriously elevated	Compensated Leydig output; hCG stimulation	Normal TT does not exclude reduced reserve; interpret with LH and hCG [7,20]
Luteinizing hormone (LH)	Elevated (compensated) or suppressed	Reduced Leydig reserve (↑ LH); hCG-driven steroid negative feedback (↓ LH)	Direction depends on hCG status [7,20]
TT/LH and cFT/LH ratios	Reduced (in hCG-negative men)	Impaired Leydig cell reserve	Research risk-stratification indices only in hCG-negative men; no standardized diagnostic cutoffs [7]
Follicle-stimulating hormone (FSH)	Frequently elevated	Germinal/Sertoli impairment; larger tumors	Marks impaired spermatogenesis [13,19,21]
Inhibin B	Reduced	Sertoli/germinal epithelium impairment	Declines further after orchiectomy [19]
Estradiol (E2)	Abnormal in ~50% (≈16% ↑, 32% ↓); higher in NSGCT	Leydig aromatization (hCG-driven); tumor production	Variable and not a standard surveillance marker [20,22]
β-hCG	Elevated in a substantial subset (esp. NSGCT)	Tumor secretion	Bioactive hCG may suppress LH; isolated low-level hCG with low TT and elevated/non-suppressed LH may reflect assay cross-reactivity [22]

Symbols: ↑ and ↓ denote a reported increase or decrease in the parameter indicated.

## Data Availability

No new data were created or analyzed in this study. Data sharing is not applicable to this article.

## Abbreviations

The following abbreviations are used in this manuscript:

ASCO	American Society of Clinical Oncology
ASRM	American Society for Reproductive Medicine
AUA	American Urological Association
CD63	cluster of differentiation 63
cFT	calculated free testosterone
CIS	carcinoma in situ
E2	Estradiol
EAU	European Association of Urology
ESMO	European Society for Medical Oncology
FSH	follicle-stimulating hormone
GCNIS	germ cell neoplasia in situ
GCT	germ cell tumor
hCG	human chorionic gonadotropin
ICSI	intracytoplasmic sperm injection
LH	luteinizing hormone
microTESE	microdissection testicular sperm extraction
NCTW	non-cancerous testicular tissue width
NDUFS1	NADH:ubiquinone oxidoreductase core subunit S1
NSGCT	non-seminomatous germ cell tumor
OS	oxidative stress
ROS	reactive oxygen species
SCSA	sperm chromatin structure assay
SDF	sperm DNA fragmentation
SHBG	sex hormone-binding globulin
TD	testosterone deficiency
TDS	testicular dysgenesis syndrome
TESE	testicular sperm extraction
TGCT	testicular germ cell tumor
TT	total testosterone
WHO	World Health Organization
